# Supplementary material for: Analysis of clinical outcomes in elderly patients with impaired swallowing function
Source: PLoS One. 2020 Sep 18;15(9):e0239440. doi: 10.1371/journal.pone.0239440 (PMC7500590; doi:10.1371/journal.pone.0239440)
Supplement: Attachment — Submitted filename: Response to Reviewers 20200819.docx [file pone.0239440.s002.docx]

PONE-D-20-09840R
Analysis of clinical outcomes in elderly patients with impaired swallowing function

We thank Reviewers #1 and #2 for recommending our paper for publication. We also appreciate Reviewer #3’s thoughtful feedback, which helped us enrich the quality of our manuscript.

Our point-by-point response is as follows:

*Reviewer #1: The authors have satisfactorily responded to the previous comments made by both reviewers. I am comfortable to recommend publication for the present form.*

We thank the reviewer for their positive comment and careful review, which helped improve the manuscript.

*Reviewer #2: The authors responded to all reviewer comments. I am happy to recommend this paper for publication.*

We thank the reviewer for their positive comment and careful review, which helped improve the manuscript.

*Reviewer #3:*

*1. L50 The speech therapist (ST) is common in Japan, but the speech language pathologist (SLP) is common in other countries. Please confirm the title.*

All speech therapists in Japan have passed the National License Examination for Speech-Language-Hearing Therapists. It is the only national certificate which officially prove the skill to engage in speech therapy in Japan and translated in English as ‘speech therapist’. We described the title of the therapists as ‘speech therapist’ because they possess the Japanese license which is not officially translated as ‘speech language pathologist’.

*2. L104 Were there any participants who constructed PEG or CV during the observation?*

Survival time of patients with PEG might be elongated by patients whose PEG was made during the observation, which suggests better health condition that did not need immediate construction of a parenteral route. Most of the patients with PEG or CV were made the parenteral nutritional route soon after their initial evaluation. Only five patients with severe dysphagia were constructed PEG 100 days or more after the initial evaluation. We analyzed survival time again after excluding those patients. The results of the analysis were the same (log-rank test: *p* < 0.05). Thus, we wish to retain the original analyses unchanged because the definition of ‘during the observation’ is not always clear.

*3. L122 The reviewer is still concerning what was the cause of dysphagia in this population. The prognosis of the disease (whether it is a progressive disease or not) is an important indicator for PEG construction. Information about the approximate cause of dysphagia of this population should be provided to the reader.*

We previously tried to divide patients by the causal disease of dysphagia such as brain infarction and by looking up the relevant ICD-10 codes. There were 801 pneumonia-related codes and 619 brain-related codes in the 912 patients. Then, we realized that a single patient often had multiple diseases, which made it difficult to presume the cause of dysphagia. Simple aging might not even be on the list of disease codes. Consequently, we focused on the direct relationship between swallowing function and survival time. For additional clarity, we added the following to the revised manuscript (page 10, lines 183-7 in the revised manuscript): “Since multiple causes of dysphagia such as senility, dementia, and cerebrovascular disease simultaneously exist in some patients, it makes it difficult to collect this information unambiguously. Among the patients with severe dysphagia (*n* = 308), the mean age was 87.2 years, and 264 patients were considered cognitively impaired.”

*4. L145 The number of deaths is 373 in the text, but it is 372 in Table 1. The number of Grade I people is 308 in the text and Table 1, but in Figure 1 it is 309.*

Thank you for your comment. We revised these errors.

*5. Table 4 "impaired cognitive function" is written twice in the legend.*

Thank you for your comment. We corrected this error.
